# A Comparison between Various Polymeric Membranes for Oily Wastewater Treatment via Membrane Distillation Process

**DOI:** 10.3390/membranes13010046

**Published:** 2022-12-29

**Authors:** Dharshini Mohanadas, Puteri Mimie Isma Nordin, Rosiah Rohani, Nur Syafiqah Farhanah Dzulkharnien, Abdul Wahab Mohammad, Peer Mohamed Abdul, Suriani Abu Bakar

**Affiliations:** 1 Department of Chemical & Process Engineering, Faculty of Engineering and Built Environment, Universiti Kebangsaan Malaysia, UKM, Bangi 43600, Selangor, Malaysia; 2 Research Centre for Sustainable Process Technology, Faculty of Engineering & Built Environment, Universiti Kebangsaan Malaysia, UKM, Bangi 43600, Selangor, Malaysia; 3 Chemical and Water Desalination Engineering Program, College of Engineering, University of Sharjah, Sharjah 27272, United Arab Emirates; 4 Nanotechnology Research Centre, Faculty of Science and Mathematics, Universiti Pendidikan Sultan Idris, Tanjung Malim 35900, Perak, Malaysia

**Keywords:** oily wastewater, membrane distillation, commercial membrane, pressure-driven membrane filtration

## Abstract

Oily wastewater (OW) is detrimental towards the environment and human health. The complex composition of OW needs an advanced treatment, such as membrane technology. Membrane distillation (MD) gives the highest rejection percentage of pollutants in wastewater, as the membrane only allows the vapor to pass its microporous membrane. However, the commercial membranes on the market are less efficient in treating OW, as they are prone to fouling. Thus, the best membrane must be identified to treat OW effectively. This study tested and compared the separation performance of different membranes, comparing the pressure-driven performance between the membrane filtration and MD. In this study, several ultrafiltration (UF) and nanofiltration (NF) membranes (NFS, NFX, XT, MT, GC and FILMTEC) were tested for their performance in treating OW (100 ppm). The XT and MT membranes (UF membrane) with contact angles of 70.4 ± 0.2° and 69.6 ± 0.26°, respectively, showed the best performance with high flux and oil removal rate. The two membranes were then tested for long-term performance for two hours with 5000 ppm oil concentration using membrane pressure-filtration and MD. The XT membrane displayed a better oil removal percentage of >99%. MD demonstrated a better removal percentage; the flux reduction was high, with average flux reduction of 82% compared to the membrane pressure-filtration method, which experienced a lower flux reduction of 25%. The hydrophilic MT and XT membranes have the tendency to overcome fouling in both methods. However, for the MD method, wetting occurred due to the feed penetrating the membrane pores, causing flux reduction. Therefore, it is important to identify the performance and characteristics of the prepared membrane, including the best membrane treatment method. To ensure that the MD membrane has good anti-fouling and anti-wetting properties, a simple and reliable membrane surface modification technique is required to be explored. The modified dual layer membrane with hydrophobic/hydrophilic properties is expected to produce effective separation in MD for future study.

## 1. Introduction

Water pollution due to human and industrial activities is one of the ongoing challenges that the world is facing today. Although the rapid growth of the industrial sector opens up economic and social opportunities that contribute to the improvement of human civilization, untreated waste that is released from this sector will adversely affect not just the environment, but also human health and ecological systems [1]. Industrial wastewater contains toxic chemicals, heavy metals, microorganisms, biological compounds, microplastics, oil and even viruses [2]. According to a report by the United Nations (UN) in 2017, 2.21 trillion cubic meters of global wastewater is discharged into the environment each year, and 2.2 billion people worldwide still lack safe and clean drinking water (UNESCO 2017). There are a few reasons why industrial wastewater is still not being treated properly by industries before releasing it to the environment, such as high operational costs, the need for a large amount of space near the industry, lack of law enforcement on discharge limits and lack of technical understanding of wastewater treatment systems [3].

In general, oily wastewater (OW) is the most critical wastewater, as it potentially threatens not only human health, but also inhibits plant growth, polluting the soil, water and air due to the harmful nature of its oil composition. The OW discharged in a large quantity from the industrial sector mainly leads to various adverse effects on the environment, such as air pollution due to the evaporation of its oil and hydrocarbon content. OW can impact clean water sources as the unfiltered contaminants flow into groundwater sources [4,5]. OW is a mixture of oil under different ranges of concentration, where it contains fats, hydrocarbon and petroleum fractions, i.e., diesel, gasoline and kerosene [4]. OW can be produced via various sources, such as industrial effluents, waste from restaurants or hotels, domestic wastewater and wastewater from agriculture. The discharge of OW is significantly increasing due to rapid industrialization, as well as the growth in population [6]. The petrochemical industry, metal cutting, paints, food and edible oil processing are the main sources of OW production. However, domestic sources such as kitchen waste, oil spills and human activities are also considered as sources of OW production [5].

There are many studies that have been conducted to separate oil from OW. Various physical, chemical and biological methods such as oil skimming, chemical oxidation, air flotation, chemical coagulation, adsorption, centrifugation and electrochemical treatment, as well as photocatalytic treatment, are performed for the separation process [1,7]. However, these techniques illustrate low separation efficiency, high operational cost, complex processes in certain cases, the formation of secondary pollutants and inefficiency in separation of oil emulsions with a droplet size of less than 10 μm [1,5]. According to Kundu and Mishra [8], the study of advanced water treatment technologies, such as membrane treatment methods, are greatly highlighted to meet discharge limits and clean water standards. Hamzah et al. [9] stated that membrane technology is the most efficient method in treating OW compared to other conventional methods. Membrane technology is effective, simple and economically viable, and can get rid of the smallest oil droplets (< 10 μm) in the OW [6,10]. The membrane separation process can be classified following its driving forces, which are pressure and temperature [9,11]. Pressure-driven membrane separation is an ultrafiltration and nanofiltration process, while thermal-driven membrane separation is a membrane distillation (MD) process [12,13]. The high-cost conventional separation process has a high consumption of energy and excessive discharge of wastewater during the process, which significantly reduces the separation/rejection efficiency. In contrast, MD involves a relatively low energy cost, minimal membrane fouling, low operating pressure and low operating temperature compared to other conventional separation techniques [14]. Therefore, MD is a promising technology to treat feed that contains oil [6,15] because it theoretically removes all the volatile compounds due to the properties of the membrane, which only allows vapor to pass through it. It can also produce highly pure water and is capable of meeting high industrial quality standards, such as in the semiconductor and pharmaceutical industries [12].

In this work, we utilized several types of commercial membranes for simulated OW treatment and analysis. Membrane wetting and membrane fouling are the main concerns of this OW analysis, as it significantly impacts the oil removal efficiency [16,17]. Initially, the commercial membranes are tested via the typical pressure-driven filtration method. The obtained high-performance membrane is then examined via the MD method to compare and determine the efficiency of the MD method. The MD technique is specifically selected for long-term performance of OW analysis, as it is a low-cost and green technology method utilizing a membrane which operates without the need of any external hydraulic pressure, and it results in high selectivity and less fouling of the membrane. The characterization and the performance comparison of the selected membranes through both methods, pressure-filtration and MD, are performed to identify a promising membrane and membrane treatment method for OW treatment. The MD process is expected to successfully demonstrate a great permeate flux measurement and significant oil removal percentage compared to the pressure-driven filtration method, indicating a promising OW separation technique.

## 2. Materials and Methods

### 2.1. Materials

NFS, NFX, XT and MT membranes were purchased from Synder Filtration. The GC membrane was purchased from GuoChu Technology, while the FILMTEC membrane was received from Dow Water Solutions. Vegetable oil was used as the oil source in the synthetic OW production.

### 2.2. Preparation of Synthetic OW

The synthetic OW mixture was prepared utilizing vegetable oil with the aim of simulating oily wastewater for the targeted treatment. OW with an oil concentration of 100 ppm was used for the membrane screening, while high-concentration OW with a concentration of 1000 and 5000 ppm were produced for the long-term membrane performance test. The mentioned concentration of vegetable oil was added into 1 L of distilled water. The mixture of oil and water was stirred at 1000 rpm for 30 min. The mixture was then sonicated for 30 min to stabilize the oil in water emulsion (O/W) and it was used right away.

### 2.3. Membrane Screening by Crossflow Membrane Filtration Method

Several types of membranes were identified using a crossflow membrane filtration method before applying them in the MD method. The type of membranes used are tabulated in Table 1.

**Figure 1:**
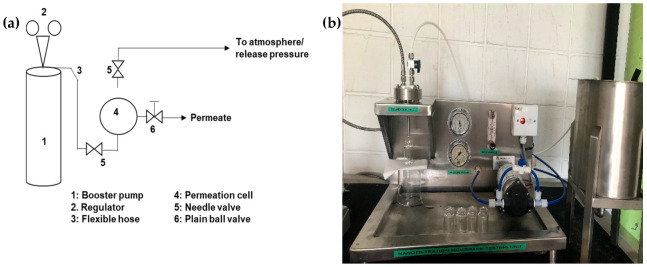
(**a**) Picture illustrates the crossflow membrane filtration setup for lab scale and (**b**) the schematic diagram of the permeation system.

The schematic diagram of the permeation system is depicted in Figure 1a, while the real crossflow membrane filtration system used to perform the test is shown in Figure 1b.

The feed pump was installed to the system to ensure continuous feed flow while the control valve was installed to control the operating pressure, which was measured by the pressure gauge. The flowmeter set was fixed at 40 L/h throughout the experiment. The membranes (Table 1) were cut in a circle with a diameter of 5.5 cm and were soaked overnight in distilled water. The membrane was then fitted into the membrane cell holder. Firstly, a compaction test was carried out using distilled water, and the test was performed at 4 bar until a stable flux value was obtained. In the clean water permeability (CWP) test, distilled water was fed in and run at a pressure of 1 bar, 2 bar and 3 bar. For each pressure, the permeate samples were collected every 1 min 5 times to measure the flux. The synthetic OW was fed into the system, and the temperature of the feed was kept constant at 27 °C using a chiller. The pressure of the membrane filtration system was kept constant at 1 bar, 2 bar and 3 bar, respectively. The permeate samples were then collected every 1 min 5 times for each of the applied pressures to measure the flux and to identify the separation membrane performance. The mentioned procedure and tests were repeated for every membrane that is listed in Table 1.

### 2.4. OW Treatment by Direct Contact Membrane Distillation

**Figure 2:**
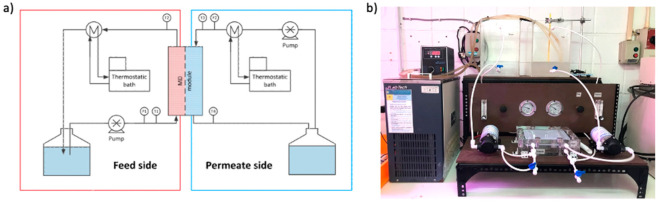
(**a**) Schematic diagram of the DCMD experimental setup and (**b**) the digital picture of DCMD setup for lab scale.

The OW treatment was carried out using direct contact membrane distillation (DCMD). The selected membranes were applied into this membrane treatment method. The DCMD system was set up as in Figure 2. Synthetic OW was fed into the feed stream of DCMD while distilled water was fed at the permeation side of DCMD. The feed and permeate stream were set to flow counter current at the membrane part, respectively. The temperature of the feed and permeate stream was set at 60 °C and 25 °C, respectively.

### 2.5. Membrane Characterization

The membrane morphology before and after the OW treatment was conducted via high-resolution field emission scanning electron microscope (FESEM, SUPRA 55VP). The surface and cross section of the membranes were analyzed via FESEM using an EHT voltage of 15 and 5 kV, respectively. The functional groups of the prepared membranes were studied via Fourier transform infrared spectrometry (FTIR) (Nicolet 6700 spectrometer from Thermo-Fisher Scientific). The drop shape analysis (DSA) (DSA100, Kruss GmbH, Germany) was performed on the proposed membrane to determine the hydrophilic properties of the sample. The average contact angle was obtained using the measurement of 3 different polar liquid (ultrapure water) droplets of about 3 μm, utilizing a 5 mm × 5 mm membrane area. The negativity of the membrane was determined using Malvern Zetasizer, as it measures the repulsion magnitude or electrostatic attraction between the membrane and the solution. The membrane was cut according to the membrane holder size and pasted onto the holder by using double-sided tape. The membrane sample and the holder were then placed inside a cuvette that contained 1 mM NaCl solution (pH 7) for the analysis.

### 2.6. Membrane Separation Performance

The separation performance of the membrane was tested using clean water permeability and oil rejection tests. Distilled water was used as the feed to measure the water flux, and it was calculated over a period of time using Equation (1) below:

(1) $$J_{w}=\frac{\Delta V}{A\Delta T\Delta P}$$

where J_w_ is the flux of the permeates (L/m^2^h.bar), ∆V is the volume of permeates (L), A is the active surface area of membrane (m^2^), ∆T is the permeation time (h) and ΔP is the trans-membrane pressure (bar). For the oil removal test, the volume of oil rejected by the membrane is determined and the oil rejection efficiency (*f* %) is calculated using Equation (2), where *C*_p_ and *C*_f_ are the oil concentration (ppm) in permeate and feed solution, respectively.

(2) $$f\left(\%\right)=\left(1-\frac{C_{P}}{C_{f}}\right)\times 100\%$$

### 2.7. OW Analysis

The concentration of OW in the feed and permeate were determined using a UV-spectrophotometer with a wavelength of 531 nm. O/W was calibrated for its different known concentrations in terms of its absorbance of the wavelength at maximum absorption. A graph of absorbance of wavelength versus oil concentration was plotted and the relationship was used to identify the oil concentration in the permeate (calibration curve is not presented here).

## 3. Results and Discussion

### 3.1. Membrane Selection for OW Treatment

**Figure 3:**
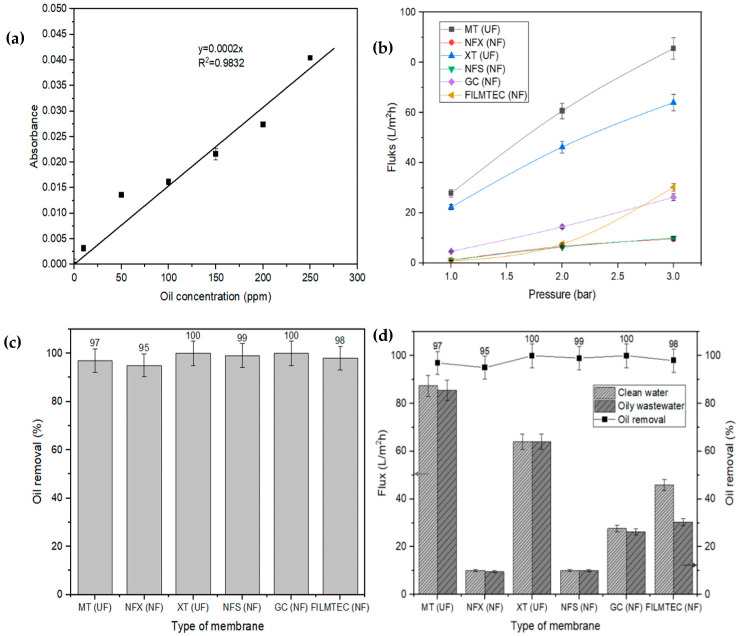
(**a**) Standard curve for oily wastewater analysis. (**b**) The permeate flux response against the applied pressure and the bar chart depicts (**c**) the performance of membranes for oil removal and (**d**) the summary of permeate flux and oil removal percentage of various filtration membranes.

Membranes on the market are not known for their performance in treating OW and usually suffer from serious fouling issues [18,19]. The oil filtration performance of ultrafiltration (UF) and nanofiltration (NF) types of membranes were analyzed and compared using a crossflow filtration method with 100 ppm oil concentration in OW. The operating pressure for the UF process is altered from 1 to 10 bar; meanwhile, it varies from 3 to 35 bar for the NF process [20]. In this study, low pressure for membrane separation process was conducted, which ranged from 1 to 3 bar. Figure 3a depicts the relationship between the absorbance of wavelength and the oil concentration, where it determines the oil concentration in the permeate. The result shows that the absorbance is directly proportional to the oil concentration, in agreement with Beer’s law. The sonification process resulted in a homogenous and physically stable OW. Since the natural properties are retained in the synthetic oil emulsion, the synthetic OW is greatly utilized for the removal of oil from water [21]. Thus, the performance of the oil separation process is not affected by the size of oil droplets in the OW. Clean water permeability (CWP) is the volume of water that passed through the membrane at permeation time and membrane area [9,22]. Figure 3b illustrates the permeate flux response of NF and UF membranes at different pressures. The result implies a linear increase in the permeate flux with an increase of the transmembrane pressure (1–3 bar). Interestingly, the MT membrane demonstrates a maximum permeate flux of 87.39 L/m^2^h at 3 bar, which is higher than the XT (64.02 L/m^2^h), FILMTEC (45.84 L/m^2^h), GC (26.12 L/m^2^h), NFS (10.08 L/m^2^h) and NFX (10.08 L/m^2^h). The tested UF membranes (MT and XT) exhibited more promising permeate flux than the NF membranes (NFX, NFS, GC and FILMTEC) because the UF membranes consist of a greater number of slightly larger-sized pores compared to the NF membranes, resulting in high CWP performance [23,24,25,26]. The distilled water generally does not have any dispersed oil particles that may block the pores of the membrane. Various membrane resistance features, e.g., adsorption, pore blocking, concentration polarization, etc., are involved in the pressure-driven processes. However, in the water flux, the membrane resistance solely depends on the pore structure of the membrane. Thus, it is proven that the difference in the MT membrane pore size plays a significant role in determining the flux performance of the membrane [27,28].

The membranes were further studied on the percentage of oil removal from OW. All the membranes depict a promising oil removal percentage, which is greater than 95%. As presented in Figure 3c, oil removal reached up to 100% for XT and GC membranes. Since XT and GC membranes can completely remove the oil content in the feed stream, this indicates high selectivity of the membranes towards the oil molecules compared to the NFS (99%), FILMTEC (98%), MT (97%) and NFX (95%).

Apart from the great oil removal percentage, the flux, membrane type and operating pressure should be considered in this application. Figure 3d illustrates the overall performance of the tested membranes. For CWP and oil removal tests, the membranes at a pressure of 3 bar demonstrate the highest flux reading, indicating the optimum operating pressure. The MT membrane illustrates a maximum flux (87.39 L/m^2^h) and a promising oil removal percentage (97%) at 3 bar. Interestingly, the XT membrane depicts 100% oil removal with good permeate flux of 64.02 L/m^2^h. Although the GC membrane achieves an excellent oil removal percentage (100%), the pore size of this NF membrane impacts the permeate flux [29]. Based on Figure 3d, the MT and XT membranes were selected as the best membranes for the MD method and the long-term OW treatment. This selection is generally based on the removal performance and the flux value of the membranes. Although the removal percentage for the MT membrane is slightly lower than the other membranes, it is still selected for further tests due to the great permeate flux. The selected MT and XT membranes are well-known UF-type membranes, which consume less energy than NF membranes, as they are cost effective and readily available in the market [30,31].

### 3.2. Membrane Characterization

**Figure 4:**
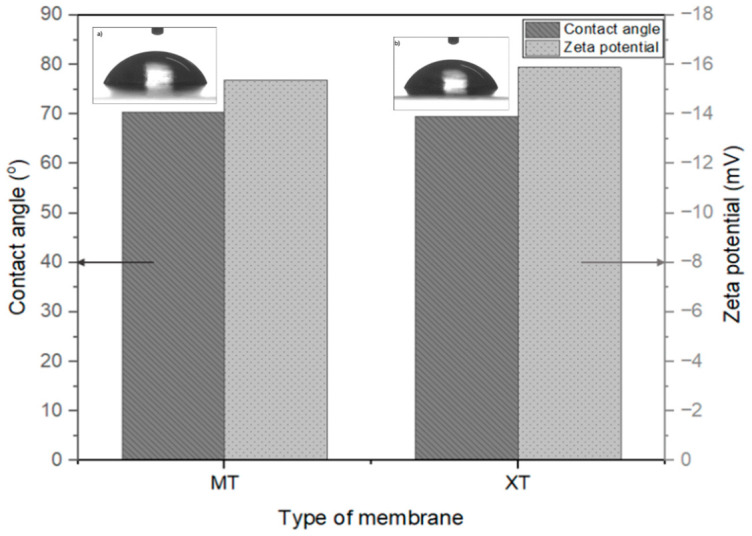
Comparison of the contact angle (inset shows the photographs of water droplet on the membranes) and the zeta potential measured for the commercial MT and XT membrane.

The surface properties of MT and XT membranes in terms of surface negativity, membrane hydrophilicity and the morphology were evaluated before the OW test. The membrane performance and anti-fouling behavior greatly affect surface hydrophilicity, considering the water contact angle [32,33]. A contact angle smaller than 90° is classified as a hydrophilic membrane, while the hydrophobic membrane produces a water contact angle more than 90° [34,35,36]. The zeta potential was used to determine the negative properties of the MT and XT membrane surface. From the inset contact angle images in Figure 4, the MT and XT membranes are hydrophilic membranes, as they depict contact angles of 70.4° ± 0.2 and 69.6° ± 0.26, respectively. The hydrophilic MT and XT membranes possess great wettability properties and can prevent severe fouling issues [37].

The zeta potential study was performed to identify the membrane surface charge and the electrostatic interaction between the membrane and contaminants [38,39]. The bar chart depicts the zeta potential of MT (−15.4 mV) and XT (−15.9 mV) membranes. The negative charge on the membrane surface is observed for both samples, as it is attributed to the presence of polar groups on its molecular chain. The charge on the surface of the UF membrane highly influences the interaction between the membrane and the particles in the feed flow. When the membrane surface and the particle have a similar charge, the rejection process takes place, reducing fouling, and concentration polarization occurs [40,41]. Since the oil droplets in the O/W emulsion are negatively charged, there is repulsion between the oil droplets and the membrane surface that lowers the fouling [42,43,44].

### 3.3. Performance of Membrane via Pressure-Filtration and MD Method

#### 3.3.1. Pressure-Filtration Technique

Although membrane technology has been recognized as an advanced separation process, the use of membranes in industry to treat OW is still limited. The fouling problems in membranes mainly occur due to surfactant adsorption, pores clogged by oil droplets and membrane degradation in the long term [45]. Different types of membrane treatment demonstrate different levels of performance in OW treatment. The performance of membrane pressure-filtration and OW method using MT and XT membranes was further compared to determine a promising OW treating method for long-term use. The polymer-type MT and XT membranes are classified under polyether sulfone (PES), denoting a polymer that has high mechanical strength and chemical stability [46,47].

**Figure 5:**
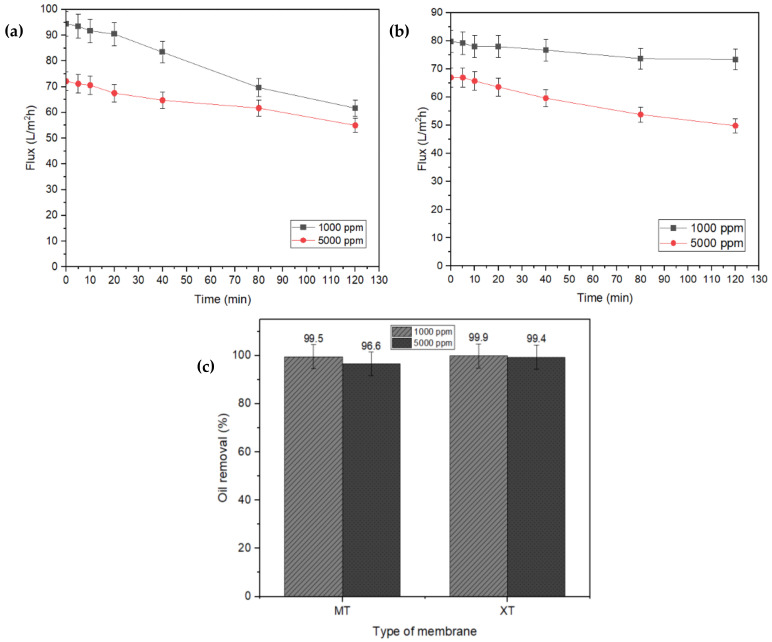
Flux performance of (**a**) MT membrane, (**b**) XT membrane via pressure-filtration method, and (**c**) oil removal percentage upon using MT and XT membrane at 1000 and 5000 ppm.

OW with an oil concentration of 1000 ppm and 5000 ppm was utilized for the pressure-filtration method. Figure 5a,b illustrates the flux performance of the MT (Figure 5a) and XT (Figure 5b) membranes in treating OW. At an oil concentration of 1000 ppm, the MT membrane recorded a maximum flux of 94.42 L/m^2^h at 0 min, which then decreased to 61.73 L/m^2^h (35% from its initial reading) after two hours of OW treatment. For the oil concentration of 5000 ppm, the flux of MT membrane dropped to 24%, which is from 72.12 to 55.00 L/m^2^h. Comparatively, the flux reading for OW with a concentration of 5000 ppm is lower than the OW with a concentration of 1000 ppm, as there is a difference in the concentration of solute in the water. Meanwhile, Figure 5b shows that the XT membrane evidenced a maximum flux reading of 79.76 and 66.92 L/m^2^h at 1000 and 5000 ppm, respectively. The flux reading of the XT membrane is noticeably lower than the MT membrane, as the molecular weight cut-off (MWCO) for the XT membrane (1000 Da) is smaller than the MT membrane (5000 Da). Considering the smaller pore size of the XT membrane, the water molecules experience difficulty passing through it. The decreasing flux for both XT and MT membranes over a period of time is primarily due to the fouling of the membrane surface.

The oil removal percentage of MT and XT membranes at 1000 and 5000 ppm were compared, as depicted in Figure 5c. Based on this figure, the oil removal percentage of the MT membrane decreased from 99.5% to 96.6% as the oil concentration increased from 1000 ppm to 5000 ppm, whereas the XT membrane only showed a slight reduction in oil removal, which was from 99.9% (1000 ppm) to 99.4% (5000 ppm). This result illustrates that the oil separation efficiency of the XT membrane is less influenced by the feed flow concentration than the MT membrane.

**Figure 6:**
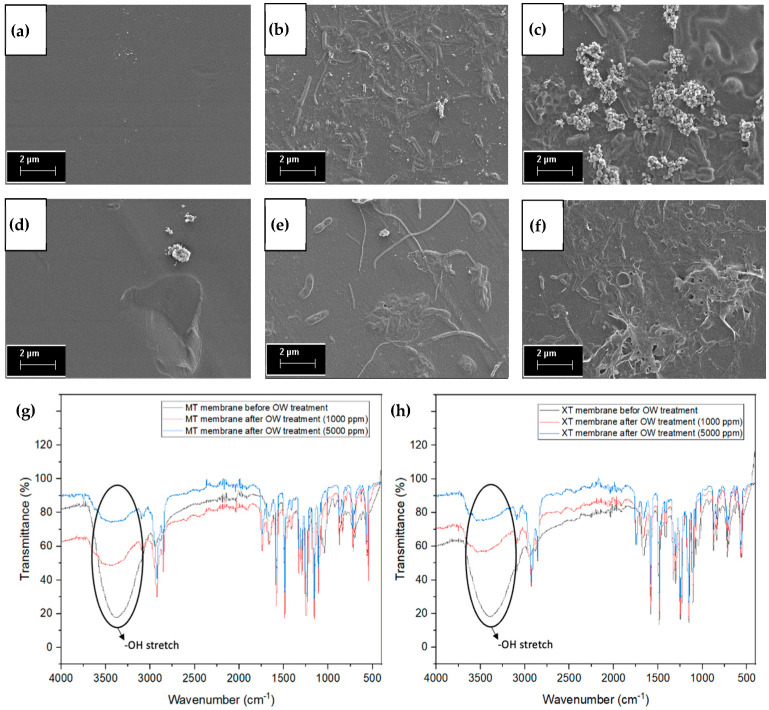
FESEM images of (**a**–**c**) MT and (**d**–**f**) XT membrane (**a**,**d**) before and after OW treatment, which takes place at the concentration of (**b**,**e**) 1000 and (**c**,**f**) 5000 ppm. FTIR spectra of (**g**) MT and (**h**) XT membrane before and after OW treatment.

Figure 6 depicts the FESEM images of the MT and XT membranes before and after the OW treatment. Both the MT (Figure 6a) and XT (Figure 6d) membranes have smooth and even surfaces before OW treatment. The smooth surface can trap contaminant particles more easily compared to membranes with a rougher surface. This is because contaminant particles tend to accumulate on the rough membrane surface, resulting in blockages and flux degradation [48,49], whereas the contaminant particles accumulate on the surface of the MT (Figure 6b) and XT (Figure 6e) membranes at 1000 ppm. This condition causes the transform pathways for the ions to be narrowed or blocked by the contaminants. The MT (Figure 6c) and XT (Figure 6f) membrane surfaces are almost/completely covered with fouling due to the two hours of OW treatment at a high oil concentration of 5000 ppm. It can be seen from the FESEM images that the biofouling occurs during the OW treatment and it is mainly caused by microorganisms. The presence of organic fouling due to oil promotes the growth of bacteria where this organic fouling acts as a food source for microorganisms (Sabri et al. 2019). The adhesion and growth of microorganisms such as bacterial cells, along with the agglomeration of extracellular material on the membrane surface, leads to biofouling [37,50,51]. Frequent cleaning and replacement of membranes are required, resulting in increased maintenance and operating costs [46].

Figure 6g,h displays the FTIR spectra of membranes before and after crossflow membrane filtration for OW with concentrations of 1000 and 5000 ppm. The overlapped MT and XT spectra demonstrate similar FTIR peaks, as both membranes are produced from a similar polymer named PES. This particular polymer provides great mechanical strength along with excellent chemical stability [18]. The -OH peak can be clearly observed at ~3400 cm^−1^ for both MT (Figure 6g) and XT (Figure 6h). The MT and XT membranes also depict -CH stretching at wavelengths ranging from 2850 to 2950 cm^−1^. The asymmetric S=O stretching within the MT and XT membrane can be detected at 1153 and 1299 cm^−1^. The peaks at 1245 and 1579 cm^−1^ are evidence of the C-O stretching and the aromatic ring stretching modes of the MT and XT membranes.

#### 3.3.2. MD Technique

**Figure 7:**
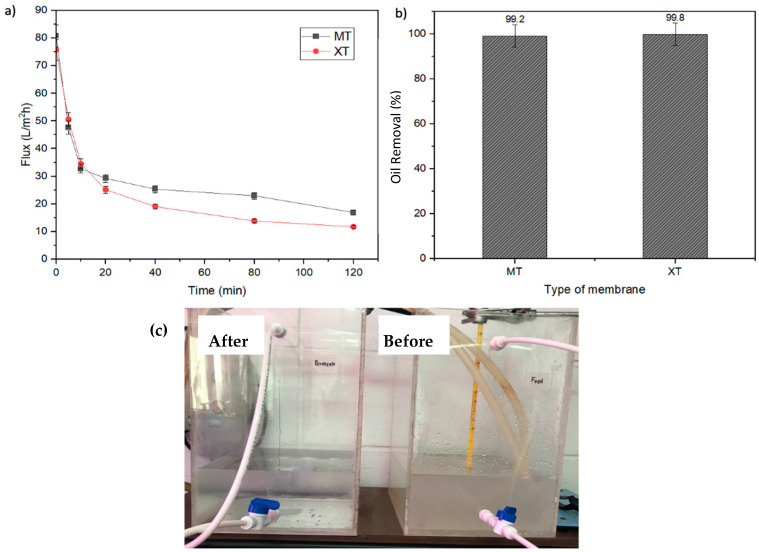
Performance of MT and XT membrane via MD method, highlighting (**a**) flux and (**b**) oil removal percentage. (**c**) The digital photographs show OW color difference before and after the MD treatment.

MD is driven by the resulting trans-membrane vapor pressure difference due to the temperature difference between the feed flow and the permeate. The MT and XT membranes were applied to the MD technique for long-term performance. The membranes and this method were tested to treat OW with an oil concentration of 5000 ppm for two hours. Figure 7a,b depicts the results for the oil removal performance of MT and XT membranes in long-term OW treatment.

Figure 7a shows the flux response for both MT and XT membranes, where a sharp decrease in flux can be seen for both membranes in the first 10 min. The MT membrane experienced a flux decrease up to 79% (from 80.78 L/m^2^h to 16.92 L/m^2^h), while the XT membrane experienced a flux decrease of 84% (from 75.68 L/m^2^h to 11.73 L/m^2^h). The flux for these two membranes is noticeably stable after 40 min. The flux deterioration occurred as there was no circulation for the permeate flow and only the feed flow underwent flow circulation. Therefore, flow instability occurred between both the feed and permeate side. This study focuses on the actual performance of the membranes without permeation flow. Therefore, the actual oil removal performance can be obtained in the permeate stream. Further studies are to be performed to test the performance of the membrane when both the feed and permeate are flowed counter current.

For the DCMD method, the flux is sensitive to the feed flow concentration [12]. Therefore, the flux decreased with increasing time due to the increase in feed flow concentration when the retentate stream was recycled to the feed. The decrease in flux was also due to the membrane wetting, where the membranes possess a hydrophilic property. The hydrophobic membrane is often used for MD to prevent wetting when in direct contact with OW [6]. However, severe fouling occurs on the membrane due to the hydrophobic bonding of the membrane surface with the oil particles [10,17]. Hence, a hydrophilic/hydrophobic composite membrane needs to be fabricated in order to produce a promising membrane for MD.

At an oil concentration of 5000 ppm (the highest tested concentration), both MT and XT membranes depict a high oil removal percentage, which is >99% (Figure 7b). This is because the MD system only allows vapor to pass through, producing very pure water [52]. Based on Figure 7c, color differences can be seen for OW before and after MD treatment. The turbid OW became very clear after MD treatment was carried out with a decrease in oil concentration from 5000 ppm to only 10 ppm.

The temperature of OW in the feed stream also influences the flux in the permeate. A higher OW temperature results in higher diffusion and mass transfer rates [20]. Temperature also affects the oil removal efficiency, as it affects the viscosity of the feed flow [53]. However, high temperature reduces the stability of the oil emulsion in water [21].

#### 3.3.3. The Performance Comparison between MD and Pressure-Filtration Method

**Figure 8:**
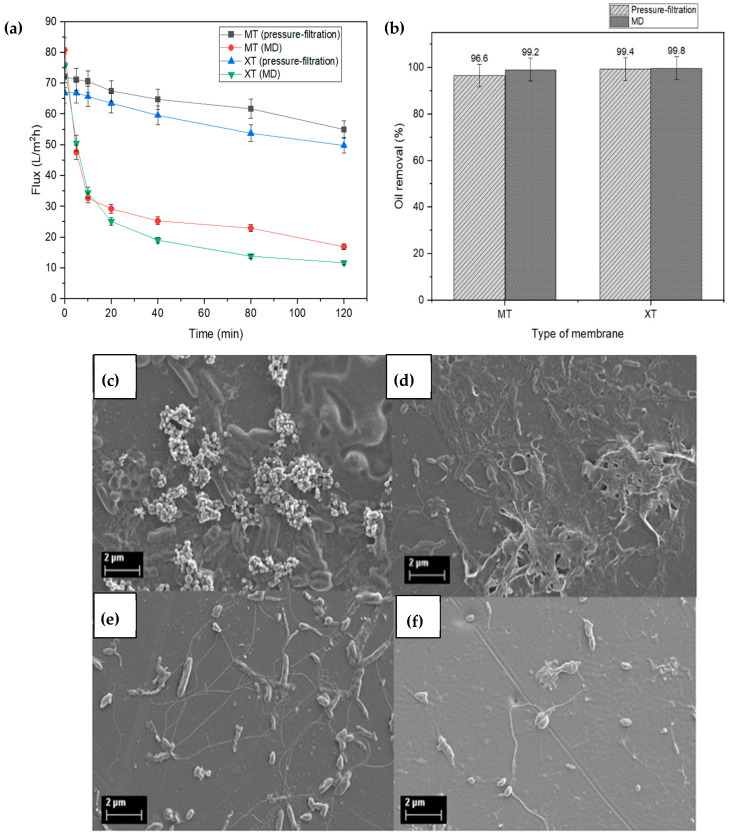
(**a**) Flux performance and (**b**) the oil removal percentage of MT and XT membranes in treating OW using pressure-filtration and MD methods. The FESEM images of MT and XT membranes after (**c**,**d**) pressure-filtration and (**e**,**f**) MD treatment.

Different membrane separation methods provide different performance in treating OW. A comparison of the performance of membrane pressure-filtration and MD is presented in Figure 8a to identify the best method in treating OW for the long term. The high flux and oil removal rate, as well as the ability of the membrane to resist fouling and wetting, are very important in selecting the best membrane method.

MD depicted a higher flux than the membrane pressure-filtration method at the beginning of the OW treatment. However, there was a sharp deterioration for MD flux due to the flow instability in the feed and permeate side because there was no flow in the permeate area. The larger active area of the MD membrane (141 cm^2^) also highly influenced the flux reading compared to the pressure-filtration membrane (79 cm^2^). Interestingly, the oil removal percentage for the MD method for both MT (99.2%) and XT (99.8%) membranes is higher than for the pressure-filtration method (MT = 96.6%, XT = 99.4%) (Figure 8b), demonstrating the efficiency of the technique for OW treatment. Hence, it is clear that the MD method is more efficient than the pressure-filtration membrane method at producing clean water.

Figure 8c–f compares the FESEM images of the MT and XT membranes after the MD and pressure-filtration methods, which was conducted at an oil concentration of 5000 ppm. The surface of the membrane depicts a different morphology after OW treatment, where both membranes that undergo pressure-filtration method are more prone to fouling issues due to accumulation of oil and microorganism growth. Comparatively, the images of the membranes used in the pressure-filtration technique (Figure 8c,d) are almost/entirely covered with organic fouling and biofouling, compared to the membranes utilized in MD method (refer Figure 8e,f. Fouling issues and growth of microorganisms were found to be limited to the membrane surfaces that were obtained from the MD operation, probably because this technique operates at a slightly high temperature. For long-term use, membrane cleaning is essentially required to restore the flux that has deteriorated due to fouling, especially in the pressure-filtration membrane method, compared to the MD method. The obtained result shows that the MD method has the most potential for OW treatment. However, the wetting problem in MD membranes with hydrophilic properties needs to be addressed in order to maintain the flux and quality of the clean water at permeate for long-term usage.

## 4. Conclusions

This study presents the evaluation of various membranes for OW treatment; a promising result was obtained for the MT and XT membranes from pressure-filtration and MD methods. The MT membrane demonstrated a maximum flux (87.39 L/m^2^h) and a promising oil removal percentage (97%) at 3 bar. Meanwhile, the XT membrane illustrated 100% oil removal with good permeate flux of 64.02 L/m^2^h. The hydrophilic-natured homogeneous MT and XT membranes with negatively charged surfaces could be clearly seen from the water droplet test and the FESEM analysis. The XT and MT membranes (UF membrane) depicted contact angles of 70.4° ± 0.2 and 69.6° ± 0.26, respectively, indicating a significant result with a high flux and oil removal rate. From the characterization analysis, it was proven that the MT and XT membranes have good anti-fouling properties against oil. The selected membranes were successfully tested for long-term use in the MD and pressure-filtration methods, and the performance was compared for OW treatment. The long-term performance was performed for 2 h with 5000 ppm oil concentration using the pressure-filtration membrane system and MD system. The XT membrane illustrated a great oil removal percentage of >99%. It was proven that the MD gave a better removal percentage; the flux reduction was high with average flux reduction of 82% compared to the pressure-filtration membrane, which experienced lower flux reduction of around 25%. The oil removal percentage for the MD method for both MT (99.2%) and XT (99.8%) membranes is noticeably higher than the pressure-filtration method (MT = 96.6%, XT = 99.4%), demonstrating the efficiency of the MD technique for OW treatment. Hence, the MD method is more efficient than the pressure-filtration membrane method in producing oil-free clean water. The performance comparison between pressure-filtration and MD illustrated that the MD with a high permeate flux at the beginning of the test faced sharp deterioration due to the flow instability that occurred between the feed and permeate flow, whereas the pressure-filtration method with a flux reading at the beginning of the test was more stable and only experienced a slight flux degradation rate. From the FESEM analysis, the membrane from the pressure-filtration method experienced worse fouling after the OW treatment as compared to the MD method. The MD membrane also faced membrane wetting issues after long-term OW treatment, which is due to the hydrophilic property. In terms of oil removal percentage, the MD demonstrated a higher removal rate than the pressure-filtration method, which is 99%. Hence, MD is a promising technique in treating OW because it can produce more pure water. In the near future, the wetting problem faced by MD can be addressed by making modifications to the surface of the membrane to produce an effective membrane for the MD technique.

## Figures and Tables

**Table 1:** Specification of the utilized membranes.

Membrane Commercial	Manufacturer	Type of Membrane	Molecular Weight Cut-Off (MWCO) (Da)/Specification	Maximum Allowable Working Pressure (MAWP)	Type of Polymer
NFS	Synder Filtration	NF	100–250	435 psi at T > 95 °C 600 psi at T < 95 °C	PA TFC
NFX		NF	150–300		PA TFC
XT		UF	1000	120 psi	PES
MT		UF	5000		PES
GC	GuoChu Technology	NF	200	-	PA
FILMTEC	Dow Water Solutions	NF	97% salt removal	-	PA TFC

## Data Availability

The data presented in this study are available on request from the corresponding author.

## References

[1] Barambu N.U., Bilad M.R., Bustam M.A., Kurnia K.A., Othman M.H.D., Nordin N.A.H.M. (2021). Development of membrane material for oily wastewater treatment: A review. Ain Shams Eng. J..

[2] Hakak S., Khan W.Z., Gilkar G.A., Haider N., Imran M., Alkatheiri M.S. (2020). Industrial wastewater management using blockchain technology: Architecture, requirements, and future directions. IEEE Internet Things Mag..

[3] Abuhasel K., Kchaou M., Alquraish M., Munusamy Y., Jeng Y.T. (2021). Oily wastewater treatment: Overview of conventional and modern methods, challenges, and future opportunities. Water.

[4] Jamaly S., Giwa A., Hasan S. (2015). Recent improvements in oily wastewater treatment: Progress, challenges, and future opportunities. J. Environ. Sci..

[5] Putatunda S., Bhattacharya S., Sen D., Bhattacharjee C. (2019). A review on the application of different treatment processes for emulsified oily wastewater. International journal of environmental science and technology. Int. J. Environ. Sci. Technol..

[6] Kalla S. (2021). Use of membrane distillation for oily wastewater treatment–A review. J. Environ. Chem. Eng..

[7] Hui L., Yan W., Juan W., Zhongming L. (2015). A review: Recent advances in oily wastewater treatment. Recent Innov. Chem. Eng. Former. Recent Pat. Chem. Eng..

[8] Kundu P., Mishra I.M. (2019). Treatment and reclamation of hydrocarbon-bearing oily wastewater as a hazardous pollutant by different processes and technologies: A state-of-the-art review. Rev. Chem. Eng..

[9] Hamzah N., Rohani R., Hassan A.R., Sharifuddin S.S., Isa M.H.M. (2018). Development of chitosan/pluronic F108/polyethersulfone (PES) nanofiltration (NF) membrane for oily wastewater treatment. AIP Conference Proceedings.

[10] Dickhout J.M., Moreno J., Biesheuvel P.M., Boels L., Lammertink R.G.H., De Vos W.M. (2017). Produced water treatment by membranes: A review from a colloidal perspective. J. Colloid Interface Sci..

[11] Bitter J. (1991). Types of Membrane Separation Processes, Mechanisms of Separation. Transport Mechanisms in Membrane Separation Processes.

[12] Drioli E., Ali A., Macedonio F. (2015). Membrane distillation: Recent developments and perspectives. Desalination.

[13] Worch E. (2019). Drinking Water Treatment: An Introduction.

[14] Onsekizoglu P. (2012). Membrane distillation: Principle, advances, limitations and future prospects in food industry. Distill. Adv. Model. Appl..

[15] Aijaz M.O., Karim M.R., Omar N.M.A., Othman M.H.D., Wahab M.A., Akhtar Uzzaman M., Alharbi H.M., Wazeer I. (2022). Recent Progress, Challenges, and Opportunities of Membrane Distillation for Heavy Metals Removal. Chem. Rec..

[16] Rezaei M., Warsinger D.M., Duke M.C., Matsuura T., Samhaber W.M. (2018). Wetting phenomena in membrane distillation: Mechanisms, reversal, and prevention. Water Res..

[17] Wang K., Hou D., Wang J., Wang Z., Tian B., Liang P. (2018). Hydrophilic surface coating on hydrophobic PTFE membrane for robust anti-oil-fouling membrane distillation. Appl. Surf. Sci..

[18] Yi G., Fan X., Quan X., Chen S., Yu H. (2019). Comparison of CNT-PVA membrane and commercial polymeric membranes in treatment of emulsified oily wastewater. Front. Environ. Sci. Eng..

[19] Zhu X., Loo H.-E., Bai R. (2013). A novel membrane showing both hydrophilic and oleophobic surface properties and its non-fouling performances for potential water treatment applications. J. Membr. Sci..

[20] Tawalbeh M., Al Mojjly A., Al-Othman A., Hilal N. (2018). Membrane separation as a pre-treatment process for oily saline water. Desalination.

[21] Medeiros A.D.L.M.D., Silva Junior C.J.G.D., Amorim J.D.P.D., Durval I.J.B., Costa A.F.D.S., Sarubbo L.A. (2022). Oily Wastewater Treatment: Methods, Challenges, and Trends. Processes.

[22] Nawi N.I.M., Ong Amat S., Bilad M.R., Nordin N.A.H.M., Shamsuddin N., Prayogi S., Narkkun T., Faungnawakij K. (2021). Development of Polyvinylidene Fluoride Membrane via Assembly of Tannic Acid and Polyvinylpyrrolidone for Filtration of Oil/Water Emulsion. Polymers.

[23] Yalcinkaya F. (2019). A review on advanced nanofiber technology for membrane distillation. J. Eng. Fibers Fabr..

[24] Marbelia L., Bilad M.R., Piassecka A., Jishna P.S., Naik P.V., Vankelecom I.F. (2016). Study of PVDF asymmetric membranes in a high-throughput membrane bioreactor (HT-MBR): Influence of phase inversion parameters and filtration performance. Sep. Purif. Technol..

[25] Marbelia L., Mulier M., Vandamme D., Muylaert K., Szymczyk A., Vankelecom I.F. (2016). Polyacrylonitrile membranes for microalgae filtration: Influence of porosity, surface charge and microalgae species on membrane fouling. Algal Res..

[26] Chung Y.T., Mahmoudi E., Mohammad A.W., Benamor A., Johnson D., Hilal N. (2017). Development of polysulfone-nanohybrid membranes using ZnO-GO composite for enhanced antifouling and antibacterial control. Desalination.

[27] Huang L., McCutcheon J.R. (2015). Impact of support layer pore size on performance of thin film composite membranes for forward osmosis. J. Membr. Sci..

[28] Yang C., Tian M., Xie Y., Li X.-M., Zhao B., He T., Liu J. (2015). Effective evaporation of CF4 plasma modified PVDF membranes in direct contact membrane distillation. J. Membr. Sci..

[29] Ong C.S., Lau W.J., Goh P.S., Ng B.C., Ismail A.F. (2014). Investigation of submerged membrane photocatalytic reactor (sMPR) operating parameters during oily wastewater treatment process. Desalination.

[30] Le N.L., Nunes S.P. (2016). Materials and membrane technologies for water and energy sustainability. Sustain. Mater. Technol..

[31] Macedo A.T.Z.N., Pulido J.M.O., Fragoso R. (2018). The use and performance of nanofiltration membranes for agro-industrial effluents purification. Nanofiltration. Lond. Intechopen Ltd..

[32] Tana J.Y., Anga W.L., Mohammada A.W. (2021). Hydrophobic Polyvinylidene Fluoride Membrane Modified with Silica Nanoparticles and Silane for Succinic Acid Purification Using Osmotic Distillation Process. J. Kejuruter..

[33] Li N., Tian Y., Zhao J., Zhang J., Kong L., Zhang J., Zuo W. (2018). Static adsorption of protein-polysaccharide hybrids on hydrophilic modified membranes based on atomic layer deposition: Anti-fouling performance and mechanism insight. J. Membr. Sci..

[34] Rohani R., Yusoff I.I., Manimaran V. (2021). Polyvinylidene difluoride-co-polyethylene glycol membrane for biohydrogen purification from palm oil mill effluent fermentation. J. Membr. Sci. Res..

[35] Hebbar R.S., Isloor A.M., Ismail A.F. (2017). Contact angle measurements. Membrane Characterization.

[36] Ahmad D., van den Boogaert I., Miller J., Presswell R., Jouhara H. (2018). Hydrophilic and hydrophobic materials and their applications. Energy Sources Part A Recover. Util. Environ. Eff..

[37] Yusoff I.I., Rohani R., Ng L.Y., Mohammad A.W. (2019). Conductive polyelectrolyte multilayers PANI membranes synthesis for tunable filtration ranges. J. Mater. Sci..

[38] Nguyen C.H., Fu C.-C., Kao D.-Y., Van Tran T.T., Juang R.-S. (2020). Adsorption removal of tetracycline from water using poly (vinylidene fluoride)/polyaniline-montmorillonite mixed matrix membranes. J. Taiwan Inst. Chem. Eng..

[39] Rho H., Chon K., Cho J. (2018). Surface charge characterization of nanofiltration membranes by potentiometric titrations and electrophoresis: Functionality vs. zeta potential. Desalination.

[40] Williams P. (2014). Membrane Charge (Zeta Potential) Effect. Encyclopedia of Membranes.

[41] Kim S., Lee S., Lee E., Sarper S., Kim C.-H., Cho J. (2009). Enhanced or reduced concentration polarization by membrane fouling in seawater reverse osmosis (SWRO) processes. Desalination.

[42] Coca J., Gutiérrez G., Benito J. (2011). Treatment of oily wastewater. Water Purification and Management.

[43] Lu D., Zhang T., Ma J. (2015). Ceramic membrane fouling during ultrafiltration of oil/water emulsions: Roles played by stabilization surfactants of oil droplets. Environ. Sci. Technol..

[44] Lu D., Zhang T., Gutierrez L., Ma J., Croué J.-P. (2016). Influence of surface properties of filtration-layer metal oxide on ceramic membrane fouling during ultrafiltration of oil/water emulsion. Environ. Sci. Technol..

[45] Al-Anzi B.S., Siang O.C. (2017). Recent developments of carbon based nanomaterials and membranes for oily wastewater treatment. RSC Adv..

[46] Manawi Y., Kochkodan V., Mahmoudi E., Johnson D.J., Mohammad A.W., Atieh M.A. (2017). Characterization and separation performance of a novel polyethersulfone membrane blended with acacia gum. Sci. Rep..

[47] Tan X.M., Rodrigue D. (2019). A review on porous polymeric membrane preparation. Part I: Production techniques with polysulfone and poly (vinylidene fluoride). Polymers.

[48] Kang S., Hoek E.M., Choi H., Shin H. (2006). Effect of membrane surface properties during the fast evaluation of cell attachment. Sep. Sci. Technol..

[49] Alias N.H., Jaafar J., Samitsu S., Matsuura T., Ismail A.F., Othman M.H.D., Rahman M.A., Othman N.H., Abdullah N., Paiman S.H. (2019). Photocatalytic nanofiber-coated alumina hollow fiber membranes for highly efficient oilfield produced water treatment. Chem. Eng. J..

[50] Al-Juboori R.A., Yusaf T. (2012). Biofouling in RO system: Mechanisms, monitoring and controlling. Desalination.

[51] Meng F., Zhang H., Yang F., Li Y., Xiao Z.J. (2006). Effect of filamentous bacteria on membrane fouling in submerged membrane bioreactor. J. Membr. Sci..

[52] Abdel-Karim A., El-Naggar M.E., Radwan E.K., Mohamed I.M., Azaam M., Kenawy E.-R. (2021). High-performance mixed-matrix membranes enabled by organically/inorganic modified montmorillonite for the treatment of hazardous textile wastewater. Chem. Eng. J..

[53] Salahi A., Badrnezhad R., Abbasi M., Mohammadi T., Rekabdar F. (2011). Oily wastewater treatment using a hybrid UF/RO system. Desalination Water Treat..

